# Supplementary figures and images for: ConFindr: rapid detection of intraspecies and cross-species contamination in bacterial whole-genome sequence data
Source: PeerJ. 2019 May 31;7:e6995. doi: 10.7717/peerj.6995 (PMC6546082; doi:10.7717/peerj.6995)

**Escherichia**

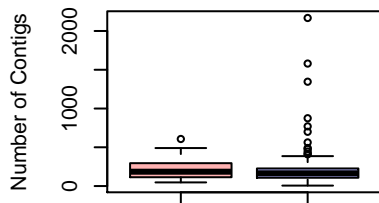

**Salmonella**

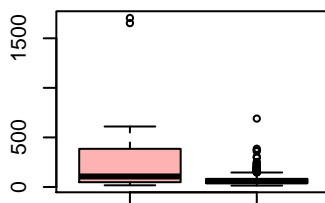

**Listeria**

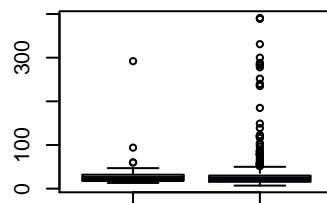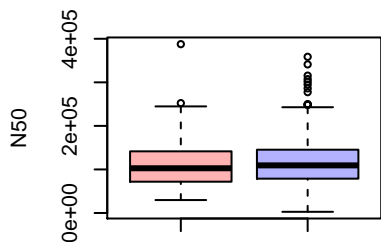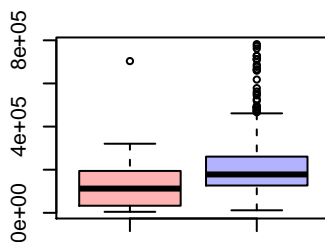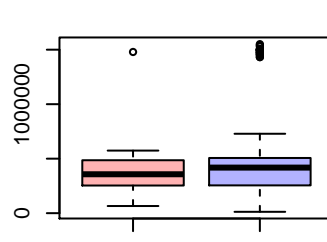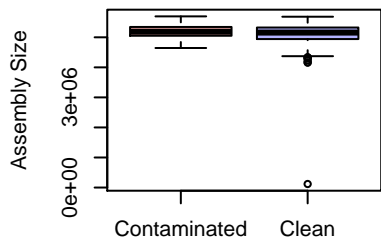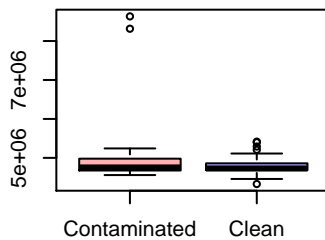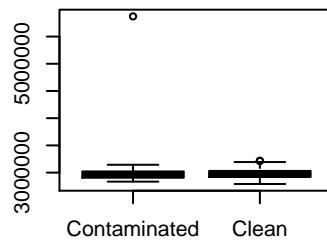

Contaminated  
Clean

Supplement: Figure S1 — Assemblies were generated for the 1,500 samples retrieved from the SRA (see Table S4). Twelve samples were excluded due to low coverage (<20×). Assembly metrics (#contigs, N50 and Genome size) for samples determined to be clean (blue) were compared to samples determined contaminated by ConFindr (red). There were 30, 7 and 17 contaminated samples for E. coli, L. monocytogenes and S. enterica, respectively. [file peerj-07-6995-s001.pdf]
